# Position in Impending Death from Chloroform

**Published:** 1869-04

**Authors:** 


### Position in Impending Death from Chloroform.

Every suggestion bearing upon the treatment of patients who exhibit alarming symptoms during the administration of chloroform, is worthy of note. Dr. Holmes, in a short communication to the Chicago *Med. Examiner*, states that in several instances in which symptoms of fatal syncope made their appearance, he found that by placing the patient with the head downwards on an inclined plane of about 40° restored the pulse at the wrist and the color to the face. He mentions one case in particular, "whose aspect was most appalling—the face and hands were cold and wet, the features pinched, the muscles of the face relaxed, lids half open, and corneæ turned upwards."

The foot of the table had not been raised fifteen seconds (the tongue having been with drawn) before the pulse was again felt and the breathing re-established. Upon restoring the patient to the horizontal position, however, the alarming symptoms re-appeared; but the change in position, by raising the foot of the table once more, was followed by relief. The Doctor thinks that by far the larger proportion of cases of death by chloroform arise from syncope, and that this may be overcome by "causing a column of blood to press upon the vessels of the brain;" and he gives the preference to the altered position, as suggested, above galvanism, artificial respiration, or other means generally adopted to restore the patient. The whole body must be inclined, not the head and shoulders alone.

—An enterprising individual has just patented a coffin in which any person unfortunate enough to get buried alive, can live for several days, and obtain nourishment from a small and convenient compartment. By using this invention, a man can open his own grave, eat a lunch, crawl out of his coffin, and drop in to see his friends on the way home.

